# Integrative Analysis of *ENAM rs3796704* Polymorphism and Eugenol–Cinnamic Acid Docking/ADMET Against Biofilm-Forming *Streptococcus Mutans*: Genetic–Phytochemical Links to Oral Dysbiosis

**DOI:** 10.3390/dj14060360

**Published:** 2026-06-11

**Authors:** Elham Hazeim Abdulkareem, Safaa Abed Latef Al-Meani, Mohammed Mukhles Ahmed, Ali Hazim Abdulkareem, Mohammed Salih Al-Janaby, Sameer Ahmed Awad, Mohammed Oday Ezzat, Saja Saadallah Abduljaleel, Zaid Mustafa Khaleel

**Affiliations:** 1 Department of Oral and Maxillofacial Surgery, College of Dentistry, University of Anbar, Ramadi 31001, Iraq; den.elham.h@uoanbar.edu.iq; 2 Department of Biotechnology, College of Science, University of Anbar, Ramadi 31001, Iraq; sc.safaa-meani@uoanbar.edu.iq (S.A.L.A.-M.); zaidmustafa683@gmail.com (Z.M.K.); 3 Department of Medical Laboratories Techniques, College of Health and Medical Technology, University of Al-Maarif, Al Anbar 31001, Iraq; 4 Department of Chemistry, College of Education for Women, University of Anbar, Anbar 31001, Iraq; 5 College of Pharmacy, University of Al-Maarif, Al Anbar 31001, Iraq

**Keywords:** ENAM gene, dental caries, *rs3796704*, high-resolution melting, polymorphism, cinnamic acid, eugenol, molecular docking, ADMET

## Abstract

**Background:** Dental caries is a chronic disease mediated by biofilm, which is caused by *Streptococcus mutans*, and enamel genetics modulates susceptibility. The variants of ENAM might alter the adhesion of enamel and bacteria. One important anti-viral target is sortase A (SrtA), which restricts colonization but does not have an impact on bacterial survival. **Aim:** The aim of this study was to find out the relationship between *ENAM rs3796704* and dental caries vulnerability among adult Iraqi Arab females and to assess the antibiofilm capacity of eugenol and cinnamic acid against *S. mutans* SrtA using molecular docking, ADMET prediction, and molecular dynamics modeling. **Methods:** A case–control study was done on 240 women (aged 25–30 years; 120 caries, 120 controls). HRM real-time PCR was done to genotype *ENAM rs3796704*. An analysis of allelic and genotypic distributions was done using chi-square tests and odds ratios (*p* < 0.05). An in silico docking analysis aimed at SrtA (PDB: 4TQX) was performed in AutoDock Vina, and this was followed by ADMET profiling and a 50 ns molecular dynamics simulation (OPLS4/TIP3P, NPT 300 K/1 atm). **Results:** The level of the G allele was found to be lower in the cases than in the controls (60% vs. 70; OR = 0.6429; *p* = 0.02), but the level of the A allele was found to be higher in the cases (40% vs. 30; OR = 1.5556; *p* = 0.02). Docking showed a minor difference in binding affinities with eugenol (−4.961 kcal/mol) and cinnamic acid (−4.939 kcal/mol) as compared with chlorhexidine (−4.692 kcal/mol). Both compounds showed stable binding for more than 50 ns as well as desirable predicted pharmacokinetics. **Conclusions:** The caries vulnerability in this sample was associated with *ENAM rs3796704*. Eugenol and cinnamic acid undergo stable dissociative interactions with SrtA and were found to have favorable safety profiles in silico. Therefore, they may be considered as adjunctive anti-virulence agents in the prevention of caries.

## 1. Introduction

Dental caries is a prevalent chronic disease that affects the teeth of a significant number of subjects of various ages because of biofilm-induced oral bacterial infection. *Streptococcus mutans* is considered to be a key cariogenic bacterium that results in the development of caries and plaque in the mouth. It generates exopolysaccharides on the tooth surface that favors cariogenic bacteria colonization and the growth of dental biofilm. This biofilm formation can be avoided by applying various antimicrobial agents like BlueM mouthwash, polypyrrole, natural flavonoids and endocannabinoid anandamide [1]. Gram-positive bacteria contain sortase, which is a cysteine transpeptidase that is essential in the covalent anchoring of surface proteins with the LPXTG motif to the peptidoglycan cell wall. Due to this feature, the surface expression of adhesins, pili assembly, host–microbe interactions, and biofilm formation depend on Sortase A (SrtA) and are, therefore, very crucial to bacterial colonization and virulence. Since it is necessary to mediate pathogenicity but not bacterial viability, SrtA has become a viable anti-virulence therapeutic target [2]. Nonetheless, irrespective of developments, there are insufficient data to elaborate on the relationship between enamel-related hereditary polymorphisms and gender-specific vulnerability in Middle Eastern populations, especially women in Iraq. The present study is novel in several key aspects. First, it represents the first investigation of the *ENAM rs3796704* polymorphism in relation to dental caries susceptibility in the Iraqi population, thereby addressing a clear geographical and ethnic knowledge gap. Second, by focusing exclusively on adult Iraqi Arab women, this study provides insight into potential gender-specific genetic contributions to caries risk—an aspect that has been largely overlooked in previous genetic association studies [3].

Importantly, this gender-focused approach is biologically justified, as previous anthropological and biomedical evidence has consistently demonstrated higher caries prevalence among females, attributed not only to behavioral factors but also to hormone-mediated alterations in saliva composition and flow across life-history stages, including puberty, menstruation, and pregnancy [4]. These physiological differences create a more cariogenic oral environment in women, underscoring the relevance of investigating female-specific genetic susceptibility.

Third, this work uniquely integrates host genetic data (ENAM polymorphisms) with in silico anti-virulence targeting of *Streptococcus mutans* Sortase A, thereby bridging human enamel genetics with bacterial biofilm-forming mechanisms. Finally, the combined use of molecular docking and ADMET profiling to evaluate the therapeutic potential of eugenol and cinnamic acid against Sortase A provides a mechanistically informed and translational framework for personalized preventive strategies in dental caries management, particularly in genetically susceptible female populations.

Dental caries is a multifactorial oral disease caused by genetic and environmental factors. It can progress due to factors such as high levels of cariogenic bacteria, poor oral hygiene, dental plaque accumulation, increased sugar intake, reduced saliva production, and the absence of fluoride exposure. It is worth noting that research conducted over the past decade has shown that, despite identical environmental risk factors, some individuals are still more susceptible to caries than others. In addition, the susceptibility to caries within specific groups remains unexplained. The data suggest that researchers have explored the possible role of genetics in susceptibility to dental caries [5,6,7].

Recent research on genetic variations, such as single-nucleotide polymorphisms (SNPs), has highlighted the significant role that genetics plays in the development of dental caries [8]. Genetic factors have been associated with the prevalence of dental caries in various populations through candidate-gene approaches and genome-wide association studies (GWASs) [9]. Many of the identified genes are involved in the development of dentin and enamel, which are crucial factors in dental caries incidence [10]. One key gene, *ENAM* (enamelin), is primarily expressed by ameloblasts during the secretory and early maturation stages and is crucial for the regulation of tooth mineralization [11]. Enamelin is the largest protein within the enamel matrix of developing teeth and plays an important role in the amelogenesis process. It also plays a key role in the mineralization and structural organization of enamel, the hardest substance in mammals [12]. Located near the mineralization front, enamelin assists in crystal elongation and organization by forming self-assembling nanostructures [13]. In the absence of enamelin, enamel crystal formation is disrupted, resulting in a disorganized mineral layer in the intercellular spaces of ameloblasts during the secretory stage [14]. The *ENAM* gene is located on chromosome 4q13.3 [15] and acts as the most significant factor in the development of normal enamel, and any change in genes that encode enamel proteins may lead to enamel malformation [11]. For instance, some mutations affect the sequence of the 32 kDa polypeptide cleavage product that is crucial for the enamelin–amelogenin interaction [16]. Weak 32 kDa polypeptides can disrupt this interaction, affecting enamel phenotype [17].

Dental caries prevalence may vary among patients despite similar environmental conditions, potentially due to genetic influences in the disease’s etiopathogenesis [18]. Recent studies have strengthened the hypothesis that genetic factors contribute to dental caries [19]. Environmental factors as well as genetic factors have been reported to play a role in the etiology of caries [20]. Amelogenesis, the process of enamel formation, involves ameloblasts—cells derived from epithelial tissue—that secrete a distinctive extracellular matrix, impacting the structure of mineralizing enamel crystallites. There are over 115 identified genetic conditions affecting amelogenesis, leading to enamel phenotypes characterized by either a decrease in enamel quantity or mineralization [19]. Enamel matrix proteins and their proteolytic cleavage fragments play an essential role in the development of enamel formation. Enamelin is one of the most important secretory enamel matrix proteins, composed of 1142 a.a, containing a 39 a.a indicative peptide [11]. It is glycosylated and phosphorylated and is rapidly cleaved into smaller fragments after secretion [16].

Mutations in the *ENAM* gene lead to amelogenesis imperfecta type 1. To date, ten mutations and 406 SNPs have been identified in the *ENAM* gene. Among the 406 SNPs identified in the *ENAM* gene, this study analyzed SNP rs3796704, located in exon 10 on chromosome 4q13.3. It is a missense variant, where the G allele is the wild type and the A allele is the minor allele [21]. This SNP was selected due to its substitution at position 763, replacing arginine with glutamine (Arg > Gln). This change can impact the protein’s size, charge, and hydrophobicity, potentially affecting enamel structure and hardness, thus increasing susceptibility to caries [22].

Previous research has validated that SNPs within the ENAM gene can influence saliva phosphorus levels [22], promote mineral loss in acidic conditions and enhance bacterial attachment and biofilm formation [23]. Research conducted in Turkey [24] and France [25] has identified a strong association between the rs3796704 polymorphism in the *ENAM* gene and dental caries.

Host genetic variability is also becoming a predictor of oral microbial community structure, i.e., the difference in caries susceptibility cannot be explained by environmental exposure and the presence of bacteria alone, as hereditary factors also determine the colonization niche on the tooth surface [26,27]. In this regard, enamel-formation gene polymorphisms, including ENAM, are conceivably capable of altering enamel microstructure and surface physicochemical characteristics and thus altering early bacterial adhesion and the consequent ecological dominance of cariogenic taxa, especially *Streptococcus mutans* (1). This host-mediated selection pressure is mechanistically consistent with findings that *S. mutans* uses cell-surface and sortase A-conjugated adhesins (e.g., SpaP and other LPXTG motif proteins) to attach to salivary components, bind extracellular matrix molecules and organize aggregation activities that facilitate stable biofilm formation [28]. All of these results suggest that a complex interplay between host genetics (including variants related to enamel) and bacterial virulence/adhesion systems determines the initial development of biofilms and the development of caries, rather than any particular factor.

Since SrtA plays a central role in biofilm-related virulence, SrtA has become a promising target of anti-virulence interventions that aim at undermining bacterial pathogenicity without causing the selective pressure that comes with bactericidal agents. Recent studies have suggested that natural phytochemicals have the potential to be safe and effective SrtA and other biofilm-related pathway inhibitors. Phenylpropanoids like eugenol, which is abundant in *Syzygium aromaticum*, and cinnamic acid, which is a hydroxycinnamate widely distributed in medicinal plants, have well-documented antimicrobial, anti-inflammatory and antibiofilm properties [29,30]. They react with bacterial cell membranes, inhibit quorum sensing and extracellular polysaccharide formation, and may disrupt enzymes in adhesion and biofilm stabilization. Computational chemistry breakthroughs have now made it possible to obtain precise characterizations of ligand–protein interactions, such as those in molecular docking, where the orientation, affinity, and binding characteristics of small molecules at enzyme active sites are predicted. Docking experiments have demonstrated that different phenolic compounds can occupy the catalytic pocket or substrate-binding groove of SrtA, preventing LPXTG motif recognition or access to the nucleophilic cysteine, which is needed to complete the transpeptidation process [31]. Concomitantly, ADMET profiling provides a good understanding of the pharmacokinetics, absorption, toxicity, and drug-likeness of phytochemicals, enabling the identification of candidates for oral therapeutics or preventive use.

While advancements in dental caries management are ongoing, studies focusing on preventive measures have yet to gain significant traction. Thus, polymorphisms in the *ENAM* gene play a crucial role in identifying caries-susceptible groups within a population, aiding in preventive dental care. To date, no research has investigated the link between ENAM gene polymorphisms and dental caries in the Iraqi population. This study aimed to investigate the relationship between the *ENAM rs3796704* polymorphism and dental caries in adult Iraqi Arab women and to evaluate the inhibitory potential of the phytochemicals eugenol and cinnamic acid against *Streptococcus mutans* Sortase A through molecular docking and ADMET analysis. The study sought to integrate clinical genotyping data with *in silico* evaluation of ligand–protein interactions in order to clarify how enamel-related genetic variation and phytochemical activity may jointly influence biofilm formation and oral dysbiosis.

## 2. Materials and Methods

**Figure 1:**
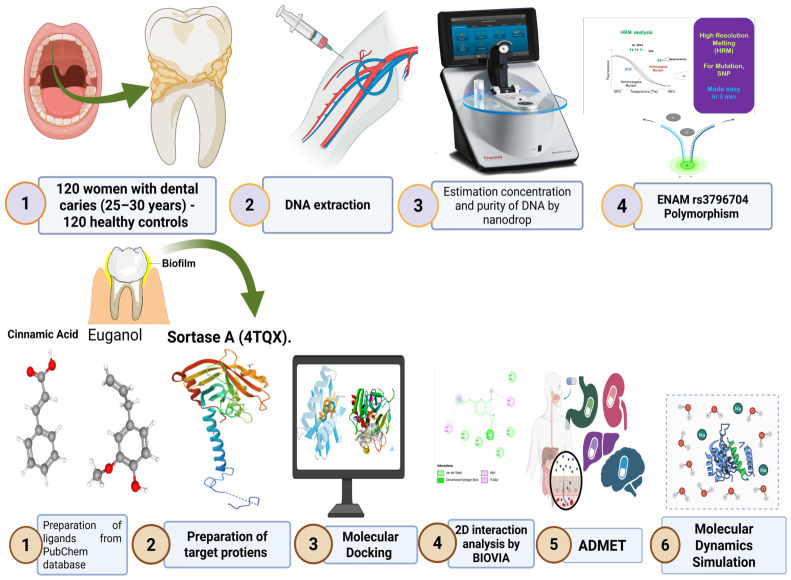
Flow chart of this study. This figure was generated using BioRender. The study design with clinical sampling and genotyping of the *ENAM rs3796704* alleles and in silico screening of eugenol and cinnamic acid with the *S. mutans Sortase A* (4TQX) target protein using docking, ADMET prediction, and molecular dynamics simulation.

This research was an interactive clinical–molecular and computational study which aimed to determine the relationship between ENAM gene polymorphism (rs3796704) and dental caries predisposition and an in vitro evaluation of a number of natural compounds that have been selected to induce *Streptococcus mutans* biofilm formation. Two hundred and forty (*n* = 240) women aged between 25 and 30 years were recruited and separated into two groups (*n* = 120): patients with dental caries and (*n* = 120) caries-free healthy controls. All of the subjects were of Arab descent; lived in Ramadi, Iraq; and had similar diets and living environments to reduce confounding variables. Genomic DNA extraction was done for all the participants using peripheral blood samples. A NanoDrop spectrophotometer (Thermo Fisher Scientific, Waltham, MA, USA) was used to determine the concentration and purity of the extracted DNA. The *ENAM rs3796704* polymorphism was genotyped by high-resolution melting (HRM) analysis that allowed the discrimination of various allelic forms. At the same time, a computational workflow was realized to investigate antibiofilm mechanisms on the molecular level. Phytochemicals representative of cinnamic acid and eugenol were picked, and the PubChem database was searched to retrieve them. The molecular target was selected as the Sortase A enzyme of *Streptococcus mutans* (PDB ID: 4TQX), as the enzyme plays a critical part in bacterial adhesion and biofilm formation in bacteria. The preparation of ligand and protein structures was performed in line with the general docking standards, and a molecular docking analysis was done to assess binding affinity and interaction patterns. Redundant docking was performed, and BIOVIA Discovery Studio version 21.1.0 (Dassault Systèmes BIOVIA, San Diego, CA, USA). was used to provide an analysis of two-dimensional interactions to describe hydrogen bonding and hydrophobic interactions post-docking. Finally, in order to identify the pharmacokinetic behavior, drug-likeness, and safety parameters of the test compounds, ADMET profiling was carried out. This combination method enabled genetic susceptibility to be correlated with the pathogenesis of dental caries at a molecular level. Women only were used to minimize sex-related biological heterogeneity and enhance cohort homogeneity in the analysis of genetic association, as shown in Figure 1.

### 2.1. Study Population and Patient Selection

From 10 September 2023 to 1 June 2024, women patients attending specialized dental clinics at the College of Dentistry, University of Anbar, were selected. They received oral and written information about the study’s objectives, methodology, and scope and signed consent forms. The study included 120 women with dental caries, aged 25 to 30, of Arab ethnicity, residing in Ramadi, Iraq, who followed the same diet and lived in similar environments, compared with 120 healthy women. Patients with genetic disorders such as amelogenesis imperfecta, dentinogenesis imperfecta, osteogenesis imperfecta, enamel dysplasia due to medications or chemicals, chronic systemic diseases, or those receiving orthodontic treatment were excluded from the study. Data for each participant were obtained through a specialized questionnaire. Patient examinations and blood collection procedures were also conducted.

Oral exams utilized a sterile dental mirror, dental probe, and dental lamp. Decay and filled teeth diagnoses were confirmed via panoramic and bitewing radiographs. Venous blood samples (2 mL) were drawn under strict aseptic conditions, collected in labelled K3 EDTA-coated tubes and stored at 4 °C until use.

Although no power calculation was conducted beforehand, the final sample size of (120 cases/120 controls) matched prior candidate-gene caries studies and was sufficient to detect moderate genetic effects under standard 8 in 100, 0.05 assumptions.

#### Dental Caries Scoring and Healthy Control

**Figure 2:**
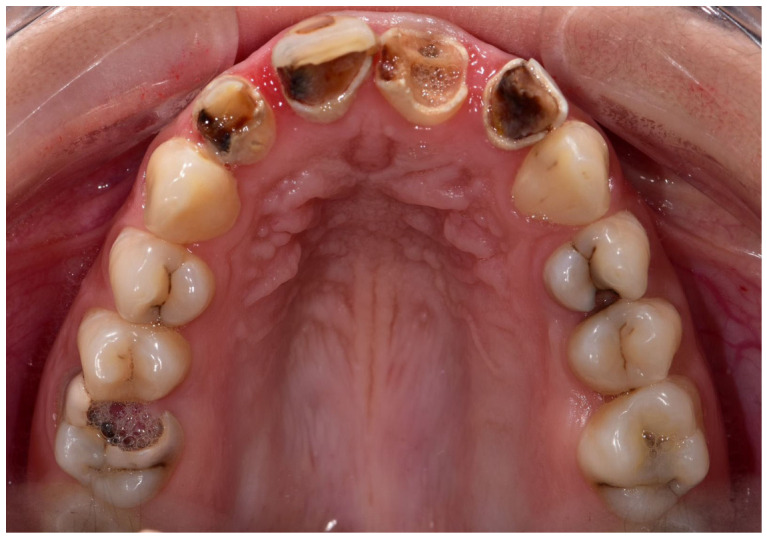
Clinical evaluation showed extreme multi-surface caries of the coronary dentition with a high level of cavitation and dentinal loss that were in keeping with the ICDAS score of 5–6. The trend indicates the presence of high caries activity and possible pulpal proximity, which are the characteristics of rampant caries presentation. The patient gave ethical approval and informed consent as stipulated by the set ethics in research.

In a bid to assure standardized and reproducible measurement of the severity of dental caries, the severity of dental caries was measured with the help of validated clinical indices that have been used in both epidemiological and genetic studies. Precisely, data on caries experience were captured as the Decayed, Missing and Filled Teeth (DMFT) index of permanent dentition as per the World Health Organization (WHO) recommendations. The DMFT index measures the cumulative burden of caries by adding together the number of decayed (D), caries-related lost (M) and restored (F) teeth, and it has been acknowledged to be both reliable and clinically significant in adults. Incidental clinical examination was conducted under standardized conditions with the help of sterile dental mirrors and explorers. Teeth were considered to be in a decayed condition when cavities or enamel/dentin softness were visible. Scoring was not included for the teeth that had been lost for reasons other than caries (e.g., orthodontic extraction or trauma) (Figure 2). The index was selected due to its frequent use in genetic association studies of dental caries, allowing comparisons with existing published data. The study was performed by a dentist, PhD (UK), majoring in Oral Health.

The level of dental caries was determined by the WHO-recommended DMFT index. The DMFT score was computed as the number of decayed (D), missing permanently carious (M) and filled permanently carious (F) teeth. Missing teeth due to causes other than caries were not counted.

The caries-free individuals (DMFT = 0) were classified as controls, and those with DMFT ≥ 1 were classified as cases.

### 2.2. Genomic DNA Extraction

To extract DNA from frozen blood samples, the Wizard^®^ Genomic DNA Purification Kit (cat. no. A1120) manufactured by the (Promega Corporation, Madison, WI, USA), was used.

Estimation of DNA concentration and purity:

The concentration and purity of the DNA were determined using a NanoDrop spectrophotometer (Thermo Fisher Scientific, Waltham, MA, USA). The purity of the A260/A280 varied within a range of 1.7 to 2.1, which is deemed to be good quality DNA to undergo downstream molecular tests.

**Figure 3:**
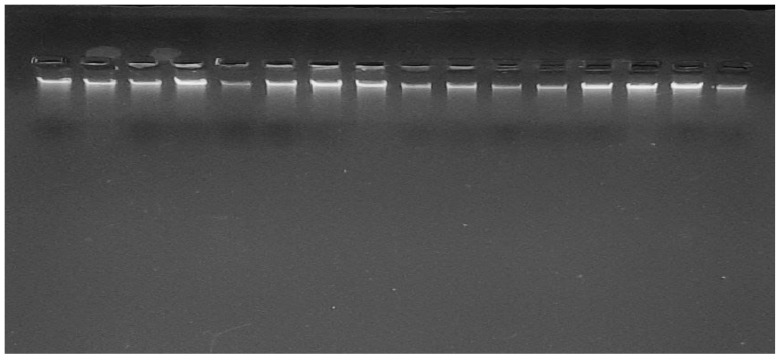
Analysis of genomic DNA samples extracted done by agarose gel electrophoresis (1% w/v). This was evaluated using the procedure of electrophoretic separation on a 1% agarose gel that had been stained with an intercalating dye and observed under UV light. The absence of smearing of discrete, high-molecular-weight bands is evidence of good DNA integrity, such that it can be used to conduct downstream molecular analyses.

Integrity of DNA:

In order to detect the quality of the bands, the extracted DNA integrity was first verified using electrophoresis in a 1% agarose gel, as shown in Figure 3. A full, uncropped image is provided in Figure S1.

### 2.3. Enamelin Gene rs3796704 SNP Genotyping

Two primers (forward and reverse) were designed for high-resolution melting (HRM) analysis after scrutinizing the ENAM gene sequence from the SNP database on NCBI. The primer characteristics are outlined in Table 1. DNA extracted from venous blood was subsequently subjected to HRM, a real-time PCR-based technique.

### 2.4. Preparing the Primers

In this study, each primer was prepared by dissolving the lyophilized sample in nuclease-free water according to the manufacturer’s instructions, and a stock solution with a concentration of 100 µM was prepared and stored at −20 °C. Diluting 10 µL of each primer stock solution in 90 µL of nuclease-free water yielded a working solution with a concentration of 10 µM, which was maintained at (−20 °C) until use.

### 2.5. HRM Real-Time PCR Runs

HRM Real-Time PCR runs were conducted using a Rotor-Gene Q Real-Time PCR System (QIAGEN, Hilden, Germany) following the program outlined in Table 2 The qPCR-HRM analysis was performed with a 0.2 °C increment from 55 to 95 °C. 2× TransStart^®^ Tip Green qPCR Super Mix (TransGen Biotech Co., Ltd., Beijing, China) was used to evaluate synthetic SNP sequences in duplicates. Triplicate synthetic controls were examined via quantitative PCR high-resolution melting (qPCR-HRM) analysis to detect allelic variations. Normalized melting curves (NMCs) and differential curves (DCs) were generated using the HRM Tool included in the integrated software (Rotor-Gene 4.4) (Rotor-Gene Q Software version 2.3.1, QIAGEN, Hilden, Germany).

### 2.6. Ligand Preparation

Eugenol (SMILES: COC1=C(C=CC(=C1)CC=C)O) and cinnamic acid (SMILES: OC(=O)C=CC1=CC=CC=C1) structures were retrieved from PubChem and converted to PDBQT format. Geometry optimization was performed using the MMFF94 force field in Avogadro 1.2. Charges and torsions were assigned with AutoDockTools 1.5.7.

### 2.7. Protein Preparation

The three-dimensional structure of *S. mutans* Sortase A (PDB ID: 4TQX) was obtained from the RCSB Protein Data Bank. Water molecules were removed, polar hydrogens were added, and Kollman charges were assigned. The active-site Cys184 was verified based on prior structural annotations. A modified structure (4tqx_modified.pdb) was used for docking.

### 2.8. Molecular Docking Protocol

Docking simulations were performed using AutoDock Vina 1.2.0 [1]. Grid box parameters were defined as follows: center: (18, 18, −2); dimensions: 20 × 20 × 20 Å; exhaustiveness: 16.

Docking of eugenol, cinnamic acid and chlorhexidine were performed as a positive control. This was recorded in kcal/mol as binding affinities. UCSF Chimera selected the highest-ranked poses to be visualized and profiled in terms of interaction.

### 2.9. Docking Validation and Controls

Chlorhexidine (MW: 505.46 Da) was used as a reference inhibitor for SrtA. Docking of chlorhexidine with 4TQX yielded an affinity of –4.692 kcal/mol, serving as a benchmark for ligand comparison.

### 2.10. ADMET Prediction

ADMET properties for eugenol, cinnamic acid, and chlorhexidine were computed using the ADMET-AI platform (version 1.3.1; Greenstone Biosciences, in collaboration with the laboratory of James Zou, Stanford University, Stanford, CA, USA, 2024) [2]. Parameters included physicochemical descriptors, gastrointestinal absorption, permeability, BBB penetration, CYP inhibition/substrate likelihood, excretion metrics, and toxicity endpoints (hERG, mutagenicity, DILI, and carcinogenicity). Results are summarized in a unified ADMET comparison table.

### 2.11. Molecular Dynamics Simulation

The simulation engine used for the molecular dynamics analysis was the Desmond simulation engine in the Maestro interface (Maestro interface, Schrodinger Release 2023-4; Schrodinger LLC, New York, NY, USA). The simulated protein–ligand complexes with the docked complexes inserted into the simulation workspace and the explicit water environment were solvated using the TIP3P model. An orthorhombic simulation cell was created, which was 10 A bigger than the outer atoms of the complex in order to fully solvate the complex.

System neutrality was obtained by adding suitable counter-ions, and physiological ionic strength was obtained by the addition of NaCl up to the final concentration of 0.15 M. The OPLS4 force field was used to describe all molecular interactions. Before the production run, the system was subjected to energy minimization, then to a staged equilibration procedure as per the default relaxation protocol given in Desmond.

The simulations of production were conducted under periodic boundary conditions in the NPT ensemble at 300 K and 1 atm temperature and pressure respectively. Temperature was controlled using the Nosé–Hoover chain thermostat, while pressure was regulated using the Martyna–Tobias–Klein barostat. The overall simulation time was fixed at 50 ns and the integration time step at 2 fs. Trajectory analysis was done at intervals of 100 ps.

Conformational stability and dynamic properties were studied with post-simulation analyses. Root mean square deviation (RMSD) and root mean square fluctuation (RMSF) were used to measure structural deviation and structural flexibility. Measurement of compactness compared to radius of gyration (Rg) was done, and the pattern of hydrogen bond formation was also measured over the lifespan of the simulation.

### 2.12. Preparation of Protein Structures Using Swiss-PdbViewer

The protein structures were retrieved in PDB (RCSB PDB, Rutgers University, New Brunswick, NJ, USA) format from the Protein Data Bank and analyzed by Swiss-PdbViewer (version 4.1) (version 4.1; Swiss Institute of Bioinformatics, Lausanne, Switzerland). The structures were all analyzed to check the absence of atoms, side chains or inconsistencies in structure. Missing atoms were rebuilt around rotamer conformations that were found in the software library, and incomplete residues were fixed with the side-chain repair tools. Hydrogen atomation was done to provide the correct valency of the molecules and stabilize the molecular structure before docking. Local energy minimization was done where required to eliminate steric interaction and poor geometries. The completed structures were exported, then used in molecular docking and molecular dynamics simulations.

### 2.13. Re-Docking Validation

The reliability of the docking protocol was evaluated with a re-docking procedure. The native ligand was removed from the crystallographic protein–ligand complex and placed back into the respective active site with the same docking parameters that were used in the initial experiments with the docking. The root mean square deviation (RMSD) was used to determine the similarity between the predicted and experimentally determined conformation. A value of RMSD less than 2.0 A was deemed to be representative of good reproducibility and methodological reliability. The calculations of re-docking were done with AutoDock Vina version 1.2.0 (Swiss Institute of Bioinformatics, Lausanne, Switzerland) with the same grid box size and scoring parameter as used in the virtual screening step.

### 2.14. Sample Size and Power Considerations

Instead of using a priori power calculation, the sample size was identified pragmatically, depending on feasibility, and the number of eligible participants which were available during the period of recruitment. We understand that this design may not be strong enough to detect minor genetic effects that are typical of SNP associations. Thus, the findings should be interpreted as exploratory and hypothesis-generating. We provide the observed effect estimates (ORs) with confidence intervals to indicate statistical uncertainty and add that replication should be done in larger and multi-center cohorts.

### 2.15. Ethical Approval

The Scientific Research Ethics Committee at the University of Anbar gave approval for the study on 12 April 2023 (Approval No. 124). Every process was done on the basis of the Declaration of Helsinki (1975, revised in 2013). Informed consent was signed by all the participants as per the standards of institutional research ethics and the principles of the Declaration of Helsinki.

### 2.16. Statistical Analysis

Statistical evaluations were performed using SPSS software (Version 26.0; IBM SPSS, Armonk, NY, USA). The assessment included testing for Hardy–Weinberg equilibrium and examining variations in allele and genotype distributions between the patient and control groups through the chi-square test. Also, odds ratios were calculated to identify alleles that would be linked to increased susceptibility or that the SNP would offer protection and the mode of inheritance of SNP analyzed was defined as dominant or recessive. A significance threshold of *p* < 0.05 was adopted for all analyses.

Associations between genotypic patterns and phenotypic outcomes were also analyzed using the same statistical platform, where chi-square testing was applied to compare allele frequencies. Molecular docking results were summarized as mean scores of the highest-ranking conformations, while ADMET predictions were interpreted descriptively to highlight differences in drug-likeness properties among the evaluated ligands.

## 3. Results

### 3.1. Genotype and Allele Frequencies

The GG genotype was less frequent in the patients (30%) compared to the controls (43.3%), suggesting a potential protective effect (OR = 0.5604, *p* = 0.1313). The GA genotype was more frequent in patients (60%) than in controls (53.3%), but this difference was not significant (OR = 1.3125, *p* = 0.4616). The AA genotype was more frequent in patients (10%) compared to controls (3.3%), suggesting a potential increased risk (OR = 3.2222, *p* = 0.1627), as shown in Table 3.

The G allele was less frequent in patients (60%) than in controls (70%), with an OR = 0.6429 (*p* = 0.02) suggesting a potential protective effect. The A allele was more frequent in patients (40%) compared to controls (30%), with an OR = 1.5556 (*p* = 0.02). These results indicate a potential increased risk, as shown in Table 4.

### 3.2. Hardy–Weinberg Equilibrium (HWE)

The HWE analysis shows that the patient and control groups were in disequilibrium, with an X^2^ value of 7.37, *p* = 0.0250, as shown in Table 5.

### 3.3. Recessive and Dominant Models for rs3796704 Association with Tooth Decay Disease

When considering the recessive model (AA vs. GG + GA), the OR was 0.3103 (*p* = 0.1627), suggesting that individuals with the AA genotype might have an increased risk of tooth decay, though the result was not significant. In the dominant model (AA + GA vs. GG), the OR was 0.5604 (*p* = 0.1313), indicating a non-significant trend towards a protective effect of the GG genotype, as shown in Table 6.

**Figure 4:**
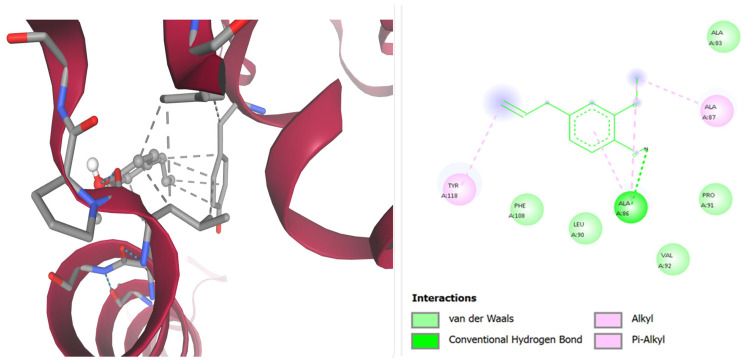
Affinity interaction between eugenol and *S. mutans* Sortase A (4TQX).

**Figure 5:**
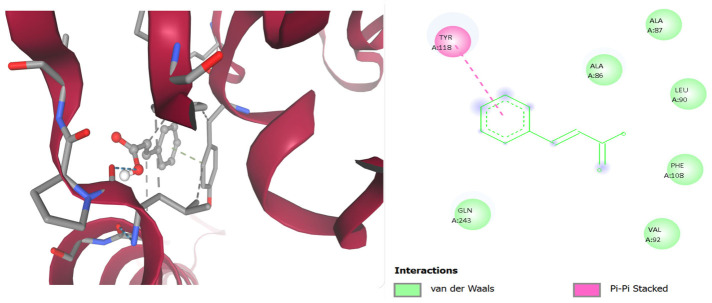
Affinity interaction between cinnamic acid and *S. mutans* Sortase A (4TQX).

**Figure 6:**
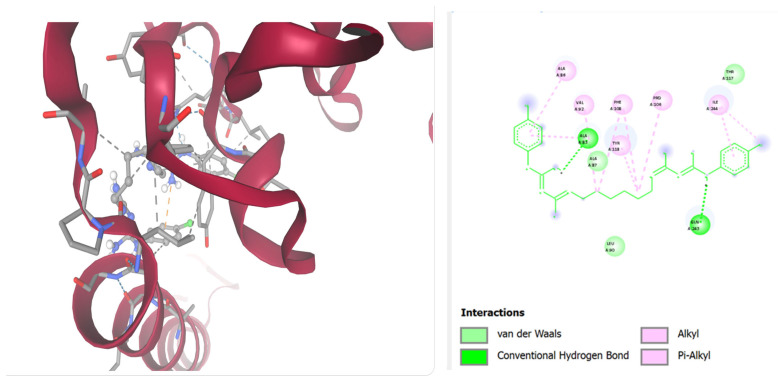
Affinity interaction between chlorhexidine and *S. mutans* Sortase A (4TQX).

Table 7 and Table 8 and Figure 4, Figure 5 and Figure 6 summarize the detailed molecular interaction landscape of eugenol and cinnamic acid within the active site of Sortase A (4TQX), revealing two distinct but complementary binding profiles that converge around key catalytic and hydrophobic regions of the enzyme.

Eugenol exhibits a native hydrophobic and aromatic-based binding pattern, which is in line with its phenylpropanoid structure. The phenolic (-OH) group of the ligand forms a stabilizing conventional hydrogen bond with Ala92, and this fixes the molecule at the entrance of the binding cleft. This residue is at the same time engaged in van der Waals interactions with the aromatic ring, which strengthens the dual anchoring effect. The allyl chain of the eugenol plays an extra role in stabilization due to the alkyl interactions with Ala97, Pro151, and Ala91, which means that the lipophilic side chain should fit through a small hydrophobic fissure covering the substrate pocket.

The phenyl group of eugenol interacts with Val166, Val168, Leu110, Phe110, and Tyr318, which are some of the main hydrophobic residues that compose the interior wall of the catalytic channel and are of the highest priority in intermolecular interactions. These residues form an apolar environment which closely grips the aromatic ring, and the eugenol is positioned parallel to the catalytic groove. This distribution is typical with ligands that replicate the hydrophobic end of natural LPXTG peptide substrates of Sortase A, indicating a competitive placement approximately at the functional core of the enzyme. Conversely, cinnamic acid has a polar–aromatic and catalytic–proximal binding profile, which is mainly due to its carboxyl moiety (-COOH) as well as planar phenyl-propenoic backbone. The ligand develops a high-affinity hydrogen bond with His120, which is a catalytic residue that is necessary in the process of cleaving the LPXTG motif. Moreover, the stabilizing polar contact is exhibited between Arg197 and the -COOH group, which means that the ligand is deeply inserted in the catalytic site. The backbone of cinnamic acid van der Waals with Gly192 and Gly193 forms part of the flexible 2-loop containing the catalytic cysteine, indicating a tight fit between the structural requirements of the ligand and the inner pocket of the enzyme. The phenyl ring is engaged in classical hydrophobic contacts with Val166, Leu169, and Ile182 and a conclusive π–π stacking interaction with Trp194, a residue that is known to stabilize aromatic substrates and inhibitors in the Sortase A binding cleft. The orientation of cinnamic acid brings the carboxyl group in close contact with Cys184 and His120, which form the nucleophilic machinery of transpeptidation, which suggests that it interferes directly with the catalytic axis of the enzyme. Together, the combined table indicates that the position of eugenol is at the hydrophobic location in the active site and that the cinnamic acid is at a deeper position with respect to the catalytic triad. This complementary binding activity indicates two mechanistic inhibitory modes: eugenol: hydrophobic channel blocker; cinnamic acid: catalytic-site interferent.

Table 7 and Table 8 presents the binding affinity values of the three evaluated ligands—eugenol, cinnamic acid, and chlorhexidine—against the Sortase A active site. Both natural compounds exhibit binding scores comparable to the positive control.

Eugenol had the highest docking score (−4.961 kcal/mol), which means that it is well-stabilized in the hydrophobic groove comprising Val166, Val168, Leu110, and Phe110. Its binding profile is optimized to be lipophilic based on its combination of hydrophobic contacts, π-alkyl interactions and targeted hydrogen bonding with Ala92, which explains its slightly increased affinity. Cinnamic acid exhibits an exceptionally similar binding affinity (−4.939 kcal/mol), although its mechanism of anchoring is more polar. Its direct interaction with his120 and arg197 and the positioning of its carboxyl group near cys184 shows that it uses the catalytic microenvironment well, and thus it can compete with the overall strength of eugenol with an alternate approach to chemistry. Chlorhexidine acts as the positive control and has a binding score of −4.692 kcal/mol, such that it is a slightly weaker binding agent than both of the plant-derived ligands. Despite its strong antimicrobial effect, the large size and rigid structure of chlorhexidine inhibit its penetration into the small catalytic groove of Sortase A, so the complementarity of chlorhexidine is to some degree lower than that of the smaller and more conformationally adaptive active natural ligands. The collective results indicate that both eugenol and cinnamic acid demonstrate significant affinity for Sortase A at energetically favorable concentrations surpassing those of the positive control. These findings support their potential as natural inhibitors of the LPXTG transpeptidation machinery in *S. mutans*.

### 3.4. Molecular Dynamics Simulation and Conformational Stability of the Cinnamic Acid–Sortase A Complex

**Figure 7:**
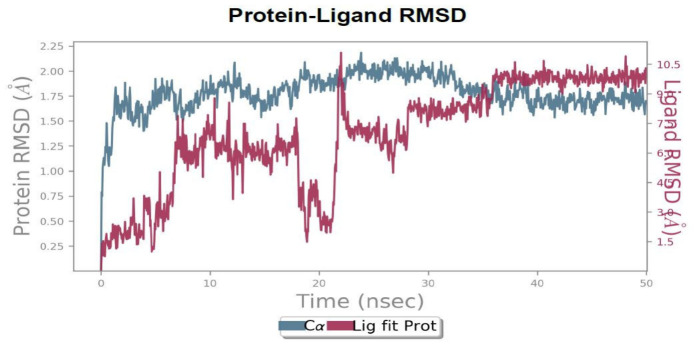
Molecular dynamics simulation and conformational stability of the cinnamic acid–Sortase A complex.

A 50 ns molecular dynamics (MD) simulation was conducted to verify the cinnamic acid–Streptococcus mutans Sortase A complex’s (PDB ID: 4TQX) structural stability and dynamics and the root mean square deviations (RMSDs) of the cinnamic acid backbone and the bound ligand were systematically analyzed. RMSD is a key marker of system equilibration, conformational integrity and persistence of binding in simulated physiological conditions, as illustrated in Figure 7.

The RMSD protein backbone also showed a rapid rise during the early equilibration period (05 ns), indicating that the required relaxation of the structure of the Sortase A en-zyme occurred in the solvated environment. After this stage, the RMSD track leveled off into a plateau with a small range of about 1.62–2.0 A for the rest of the 50 ns simulation. The presence of such a stable RMSD profile would suggest that sortase A main-bound has no significant conformational rearrangements and would confirm that Sortase A main-bound is a structurally conserved fold during the simulation, thus supporting the high intrinsic stability of the protein in complex with the ligand.

Simultaneously, ligand RMSD analysis that evaluates ligand binding to the protein backbone through the fitting of the cinnamic acid molecule showed an initial adaptive phase with moderate variations throughout the initial period of the simulation. These changes are linked to the ligand reorientation and optimization of intermolecular contacts in the binding pocket. Notably, following some 25–30 ms, the ligand RMSD had stabilized and was held steady all the way to the conclusion of the simulation with no signs of ligand displacement or dissociation. This stabilization shows that the cinnamic acid was stabilized in a good binding conformation and was firmly bound in the Sortase A active site.

It is worth noting that the stabilization patterns were synchronous between protein and ligand RMSD profiles, indicating the presence of a well-equilibrated and dynamically stable protein–ligand complex. This coupled stability is an indication of high intermolecular compatibility and helps the maintenance of non-covalent interactions that control the retention of ligands. Mechanistically, the long-term stability of the RMSD of the simulation (50 ns) is a strong indication of the reliability of the docking predictions and indicates that cinnamic acid is a strong stabilizer of the binding pocket of Sortase A.

The MD simulation results, in general, indicate that cinnamic acid is dynamically persistent in forming a conformationally stable complex with *S. mutans* Sortase A. These results offer powerful computational arguments as to why cinnamic acid is a potential Sortase A inhibitor, and thus underline its potential application in natural anti-virulence against bacterial adhesion and biofilm formation. More quantitative information on the contributions to binding free energy can be obtained through further energetic calculations, including MM-PBSA/MM-GBSA calculations.

### 3.5. Molecular Dynamics Simulation and Stability Assessment of the Eugenol–Sortase A Complex

**Figure 8:**
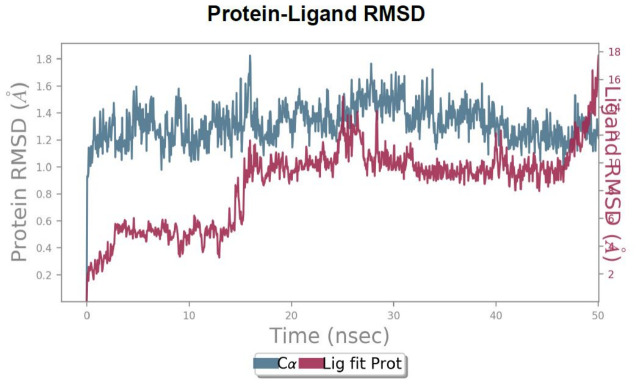
Molecular dynamics simulation and stability assessment of the eugenol–Sortase A complex.

Figure 8 illustrates the conformational and dynamic stability of the eugenol Streptococcus mutans Sortase A complex (PDB ID: 4TQX), which had been extensively tested using a 50 nm molecular dynamics (MD) simulation. The protein backbone (Cajor atoms) and ligand RMSD trajectories were used to study the equilibration of the system, structural stability, and binding stability in solvated near-physiological conditions.

A sudden increase in the protein backbone RMSD was observed at the first equilibration stage (02 ns), and then it stabilized at the interval of about 1.21.6 A most of the time during the simulation. This initial change is an indication of relaxation of the Sortase A structure when it is solvated and at the same temperature. Notably, there was no long-term drift in the RMSD or sudden change in RMSD over the 50 ns trajectory, meaning that sortase A was in a very stable global fold when in the presence of eugenol, which implies that the binding of the ligand did not cause any destabilizing conformational changes.

Re-evaluation of multi-phase behavior was detected by Ligand RMSD analysis, after binding eugenol to the protein backbone. In the initial stage of simulation (015 ns), the ligand RMSD rose slowly, as a result of the initial reorientation and optimization of intermolecular interactions in the binding pocket. There seems to be a short-lived rise in ligand RMSD around 1525 ns, indicating localized changes in the binding site probably by adaptive side-chain motions and by solvent-mediated interactions. After this stage, the ligand RMSD reached a steady point and did not change significantly over the rest of the simulation period (around 25–45 ns), suggesting that the ligand was bound to the active site. The gradual increase at the final stages of the simulation was not accompanied by the destabilization of proteins, which indicated the presence of conformational flexibility but not dissociation with the ligand.

These similarities in the stabilization patterns of both protein and ligand RMSD pro-files are a strong indicator of a dynamic equilibrium between a protein and a ligand com-complex. The fact that eugenol is able to remain in a constant relationship with Sortase A regardless of minor adaptive changes indicates the desirable compatibility of binding and stability of the interaction in dynamic environments. This is typical of biologically relevant ligand binding; moderate flexibility can be used to promote interaction persistence without affecting complex stability.

On the whole, the 50 ns MD simulation presents a convincing piece of evidence that eugenol is a structurally stable and dynamically persistent complex with *S. mutans* Sortase A. Such results confirm the dock results and indicate that eugenol is a good stabilizer of the Sortase A binding milieu, supporting its possible use as a natural anti-virulence factor against bacterial adhesion and biofilm-related pathogenicity. Additional mechanistic understanding of the energetic contributions that control this interaction would be obtained with further free energy calculations and residue-level fluctuation analyses.

According to a molecular dynamics simulation of 50 ns, eugenol is more dynamically stable and exhibits better binding adaptability to Streptococcus mutans Sortase A than cinnamic acid. The reduced and more stable protein RMSD, combined with the ability to retain the ligand over time and adaptability, show that eugenol forms a more dynamically advantageous and stable complex.

Table 9 shows the ADMET analysis, which demonstrates significant disparities in the physicochemical and pharmacokinetic profiles of eugenol, cinnamic acid and chlorhexidine because of the differences in their chemical structure. Eugenol and cinnamic acid have low-molecular-weight phytochemicals (164.20 and 148.16 Da) and chlorhexidine represents a phytochemical with a much higher molecular mass (505.46 Da), which is a determining factor in their permeability, distribution, and stability in the body. Both eugenol and cinnamic acid are entirely compliant with Lipinski’s Rule of Five, which is indicative of drug-likeness and good oral absorption, and chlorhexidine meets only two out of four parameters because of its size and the large number of hydrogen bond donors (*n* = 6). Polar shift between the series can be observed in the TPSA values, where the small polar surfaces of eugenol (29.46 A 2) and cinnamic acid (37.30 A 2) contrast highly with the large surface area of chlorhexidine (177.58 A 2), which leads to its lower rate of membrane penetration and partial absorption in the intestine. Both eugenol and cinnamic acid had the highest predicted human intestinal absorption (1.00), but chlorhexidine had a smaller yet relatively high value (0.85). The highest oral bioavailability was recorded for cinnamic acid (0.92), medium bioavailability was recorded for chlorhexidine (0.66), and a relatively low bioavailability was recorded for eugenol (0.44), which is in line with the interaction between lipophilicity, solubility, and TPSA. According to the aqueous solubility profiles, cinnamic acid, eugenol, and chlorhexidine are moderately soluble, but chlorhexidine is less soluble (–3.40 log mol/L). The measurements of passive permeability (cellular and PAMPA) again confirm that eugenol diffuses readily across lipid bilayers, and chlorhexidine exhibits limited transcellular diffusion, which is consistent with its size and polarity. Distribution parameters were also used to discriminate even more between the molecules. Eugenol and cinnamic acid have good BBB penetration (0.77 and 0.80) in line with their physicochemical properties, while chlorhexidine has poor BBB penetration (0.59). Cinnamic acid (92.79%), chlorhexidine (84.67%), and eugenol (80.45) show the highest plasma protein binding rates, and this affects their free drug fractions and systemic availability. The results for the volume of distribution show that the distributions of eugenol (0.00 L/kg), cinnamic acid (4.34 L/kg), and chlorhexidine (22.55 L/kg) are progressive, with minimum tissue retention of chlorhexidine. The metabolites show that eugenol has a broader interaction with CYP isoforms, especially CYP1A2 (0.67) and CYP2C19 (0.46), and cinnamic acid has very low interactions with all enzymes. Chlorhexidine exhibits significant inhibition of CYP2D6 (0.83), indicating possible metabolic liabilities. Substrate probabilities suggest that chlorhexidine is more likely to be metabolized by CYP3A4 (0.71) than the plant-derived compounds. Molecular size and stability have a significant impact on excretion characteristics. Eugenol and cinnamic acid show negligible predicted half-lives (0.00 h), which is a sign of rapid clearance, whereas chlorhexidine displays a long half-life (55.73 h), which is a sign of slow clearance and high metabolic stability. The hepatocyte and microsomal clearance values support this pattern, where cinnamic acid has high hepatocyte clearance and eugenol has high microsomal clearance, whereas chlorhexidine has moderate clearance in the two systems. Toxicity profiling demonstrates a positive safety trend for eugenol and cinnamic acid and a more negative safety profile for chlorhexidine. Chlorhexidine has a high hERG channel-blocking potential (0.90), which indicates a cardiotoxic potential, while eugenol and cinnamic acid have a low potential (0.07 and 0.003). The likelihood of mutagenicity and carcinogenicity increases from cinnamic acid to eugenol, with chlorhexidine exhibiting the highest rates due to its greater biological reactivity. The predicted interactions of chlorhexidine with nuclear receptors and stress pathways, such as disruption of the mitochondrial membrane potential (0.18) and p53 engagement (0.15), are stronger than the interactions of the plant compounds. The lowest acute toxicity (LD50) was recorded for cinnamic acid (1.60 log(1/mol/kg)), followed by chlorhexidine (2.53), and the highest toxic load occurred at higher doses. On the whole, the comparison of the ADMET results shows the difference between small, orally bioavailable phytochemicals (eugenol and cinnamic acid) and the larger, cationic antiseptic agent chlorhexidine, which exhibits lower permeability, longer retention, and greater toxicity indices. All these parameters indicate the differences in the pharmacokinetic and safety factors of each compound.

Table 10 demonstrates a comparative target profile. Table 8 is especially significant regarding dental biofilm, wherein inhibition of Sortase A (SrtA) in *Streptococcus mutans* is shown to be key to preventing the anchoring of surface adhesins and inhibiting the initial formation of plaque. Out of the tested compounds, eugenol is shown to have interactions with a variety of signaling and metabolic targets consistent with previously reported antibacterial and anti-adhesive activity, and cinnamic acid is shown to have a high affinity to HCAR2 and various carbonic anhydrase isoforms, which is consistent with its reported ability to alter microbial metabolic activity and disrupt biofilm integrity. On the other hand, chlorhexidine exhibits a narrow predicted target spectrum (as expected of its broad and non-membrane-disruptive activity). These differences emphasize the mechanistic heterogeneity of the compounds and the potential of eugenol and cinnamic acid to be used as supplementary agents to disrupt SrtA-dependent adhesion and the formation of *S. mutans* dental biofilm.

## 4. Discussion

Numerous studies have demonstrated that the prevalence of dental caries is higher in women than in men. In a study of a Swedish Early Modern population (1500–1620) (society), according to skeletal evidence found in a local graveyard in Gamlestaden, Gothenburg, dental pathological lesions were extremely widespread, and women had greatly more caries experience than men [4]. In a study carried out in the College of Dentistry, the University of Babylon, the authors identified the prevalence of dental caries in women compared to men, with region of residence playing a significant role in caries experience [32]. Another study done clinically in Bangladesh revealed that the prevalence of dental caries was higher in females (57) than in males (43). Another point that the authors made is that caries was mostly distributed on the occlusal surfaces rather than the proximal surfaces, with gender- and surface-specific differences in caries distribution [33].

Such sex-related differences can be attributed to biological factors, including hormonal changes and salivary variations in composition and flow rate, not to mention dietary and socio-cultural factors that might have predisposed females to dental caries.

*ENAM rs3796704* polymorphisms are missense variants that substitute guanine (G) with adenine (A) and change the [CGG] genetic code for arginine (Arg) to [CAG] for glutamine (Gln) in the coding sequence. Naturally, each amino acid has its characteristics and role in the peptide chain. Changing the amino acid at any site in the chain with another amino acid will lead to a change in the characteristics of the peptide chain, and thus the function of the protein in general will be affected. Rajpar et al. proved that an enamelin splicing mutation is the cause of an autosomal-dominant type of amelogenesis imperfecta, which is evidence that structural defects in enamelin can directly affect enamel formation and integrity [34]. These results favor the biological possibility that ENAM polymorphisms, such as *rs3796704*, can affect enamel structure and possibly could be used to modify vulnerability to dental caries [35]. In this case–control study on *ENAM rs3796704* polymorphisms, it was associated with the risk of dental caries in women of rural ethnicity in Iraq.

High-resolution melting (HRM) analysis, a modern post-PCR method for identifying nucleic acid sequence variations, was utilized to genotype the enamelin gene rs3796704. The method detects subtle differences in the melting curves of PCR products [36].

Variations in the ENAM gene heighten susceptibility to dental caries through different mechanisms. Earlier research indicates that ENAM gene disparities may result in increased mineral loss from enamel in acidic environments [37]. Alterations in the ENAM gene may favor the deposition of biofilm and attachment by bacteria. Also, ENAM gene alterations may disorganize the saliva phosphorus level, which affects the enamel remineralization [38]. The evaluation of the relationship between the SNP of the ENAM gene, SNP rs3796704, and dental caries in the current study was not consistent with that of Chaussain et al. [39].

In one study, it was possible to find that the polymorphism of the rs3796704 does not have a direct impact on dental caries in Turkish children, but possible interacts with Streptococcus mutans. However, another study has found that this SNP of the ENAM gene, in combination with environmental factors, could not be related to early childhood caries in Turkish children, which is contrary to the current findings [40]. In another study, it was found that the dental caries disease is not the only disease connected with the presence of the rs3796704 but it has a big influence on the development of the molar incisor hypomineralization (MIH). These results indicate that the presence of the G allele in this SNP increases the predisposition of the individuals to the occurrence of hypomineralization in individuals 17 times in comparison with the presence of the A allele [41].

These clinical–genetic results were furthered analyzed in silico by assessing *S. mutans* Sortase A (SrtA), which is a proteolytic enzyme needed to anchor LPXTG-containing adhesins that facilitate initial surface colonization and biofilm maturation [1]. Molecular docking showed that eugenol and cinnamic acid have affinities with SrtA that are higher than those of the commonly used antiseptic chlorhexidine. Although the change in absolute values of docking is small, the interaction profile of qualitative interaction provides important mechanistic information. Eugenol interacts with the hydrophobic channel of the catalytic channel to establish a stabilizing hydrogen bond with Ala92 and long-range hydrophobic and π-alkyl interactions with Val166, Val168, Leu110, Phe110 and Tyr318. These residues constitute the hydrophobic wall around the substrate-binding groove, and inhibition of this area is known to prevent access of LPXTG peptides, as well as the enzyme activity [42,43]. This stance implies that eugenol can be used as a hydrophobic channel blocker that has the possibility to interfere with the initial phases of *S. mutans* adhesion.

The binding pattern of cinnamic acid is also different, with the carboxyl group facing His120 and the catalytic cysteine (Cys184) directly. The ligand is in close proximity to the SrtA catalytic dyad, as a result of hydrogen bonding with His120 and other polar stabilizations by Arg197. The aromatic ring also stabilizes the binding by pi-pi stacking with Trp194 and hydrophobic interactions with Val166, Leu169 and Ile182. These patterns of interaction are in line with known competitive inhibition mechanisms of cysteine-dependent transpeptidases, where the occupancy of the catalytic pocket by ligands blocks nucleophilic attack of the LPXTG motif [42,44]. Therefore, cinnamic acid acts as a catalytic-site interferent, which directly inhibits the residues needed in the process of transpeptidation.

The difference in the complementary binding of both phytochemicals is that eugenol primarily interacts with the hydrophobic access channel and cinnamic acid interacts with the catalytic nucleus, indicating the possibility of additive or synergistic inhibition of SrtA. This dual mechanism could be beneficial in disrupting the multistep assembly of *S. mutans* surface adhesins, thereby reducing biofilm cohesion and virulence.

The docking score of chlorhexidine (CHX) with regard to *S. mutans* Sortase A was only moderate in the Vina simulations conducted in this study, but it can serve as a benchmark in a purely clinical context. CHX is commonly accepted as the gold-standard chemical antiplaque agent in the dentistry field, and it is generally used as a comparator reference point when novel antibiofilm interventions are being tested [45]. Mechanistically, CHX works by cationic membrane-active antimicrobial action and high substantivity, i.e., adsorption on pellicle-covered enamel and oral tissues, which allows it to be active long after rinsing [46]. Since these prevailing in vivo actions need not involve high-affinity binding to the comparatively small SrtA catalytic groove, a mediocre predicted docking score is feasible. Thus, CHX was applied in the present study as a clinically familiar positive-control comparator and not as a SrtA-specific inhibitor, thus permitting the binding of eugenol and cinnamic acid to be explained in comparison to a standard oral antiseptic. Other similar modeling studies have also used CHX as a reference control in SrtA docking comparisons [47].

ADMET predictions further support the feasibility of eugenol and cinnamic acid as adjunctive antibiofilm agents. Both compounds showed good intestinal absorption, moderate oral bioavailability and toxicities that were much more acceptable than chlorhexidine, which is known to cause mucosal irritation and dysgeusia and ecological disturbance of the oral microbiota with extended use [42]. Their physicochemical characteristics, moderate lipophilicity and low polar surface area and lack of strong hERG inhibition in-vitro point to their compatibility with topical oral preparations like varnishes, gels and rinses.

Combining the genetic and docking data, a consistent model is formed, according to which carriers of the ENAM rs3796704 A allele introduce into the body enamel surfaces that are more prone to the loss of minerals, as well as those more favorable to bacterial adherence. This susceptibility is further increased by *S. mutans* SrtA operation that aids in the firm anchoring of adhesins. Here, the prevention of caries in genetically predisposed individuals with the inhibition of SrtA by either eugenol or cinnamic acid can provide a specific approach to caries prevention due to the structural weakness dictated by the ENAM variant.

This study is enhanced by the in silico part, which analyzes *Streptococcus mutans* Sortase A (SrtA), the cysteine transpeptidase needed to anchor the surface-binding adhesins of LPXTG, which direct surface binding and biofilm formation in the bacterium [48]. Molecular docking indicates that the affinities of eugenol and cinnamic acid in binding are greater than that of chlorhexidine, which is the standard antiseptic used orally at present. The statistical discrepancy in the affinity values is still relatively small, but the patterns of interaction give obvious mechanistic definitions. Eugenol was placed deep in the hydrophobic section of the SrtA active site. It created a stabilizing hydrogen bond with Ala92 using its phenolic hydroxyl group and made multiple π -alkyl and van der Waals contacts with Val166, Val168, Leu110, Phe110, and Tyr318. These residues form the hydrophobic wall that encloses the LPXTG-binding pocket, which has been found to block its entrance and SrtA activity when it is blocked [49]. This profile of interaction shows that eugenol is probably a hydrophobic channel blocker that will lower the access of the natural substrate to the catalytic groove. Cinnamic acid exhibited a specific catalytic–proximal binding mode. Its carboxyl group is directly hydrogen-bonded with His120 and supplemented with another polar interaction with Arg197. The ligand backbone bound Gly192 and Gly193 in the flexible 20-residue 2-loop of the Cys184 catalytic site. The cinnamic acid aromatic ring was found to be in hydrophobic contact with Val166, Leu169, and Ile182 and π-π tacking with Trp194. The orientation of the carboxyl group towards His120 and Cys184 is also in line with the known competitive inhibition mechanism of cysteine-dependent transpeptidases, wherein a blockage of the catalytic pocket prevents a nucleophilic attack on the LPXTG motif [49].

Together, the two phytochemicals demonstrate complementary mechanisms of interference with SrtA: eugenol blocks the hydrophobic access channel, and cinnamic acid targets the catalytic center. Plants naturally synthesize a wide range of bioactive compounds that have great therapeutic potential. The rise in synthetic additive worries has elevated the demand for natural and plant-based medicines [50]; their phytochemical compounds allow herbals to be mighty regulators of host-specific cellular activities, such as immune reactions, mitosis, apoptosis, and signal transduction [51].

Eugenol has been reported to exert significant antibacterial and antibiofilm activities against periodontal pathogens. Specifically, Zhang et al. revealed that eugenol obtained with Syzygium aromaticum essential oil had strong inhibitory activities against planktonic Porphyromonas gingivalis, and membrane disruption was found to be the main antibacterial mechanism of action. The SEM showed cellular shrinkage and lysis, whereas permeability tests indicated irreparable damage to the bacterial plasma membrane. Additionally, eugenol had a significant effect of inhibiting biofilm formation during the early developmental phase and decreased preformed biofilms. This antibiofilm effect was linked to the down-regulation of important virulence genes of adhesion and biofilm development, such as fimA, hagA, hagB, rgpA, rgpB and kgp, in a mechanistic manner. The results also confirm that eugenol is a promising therapeutic aid for oral infections because it has therapeutic potential as an agent against biofilms of natural origin and justify its use in oral infection control systems [52]. There is recent evidence that cinnamic acid could be a promising antibiofilm agent against complex oral biofilms. In a study by al-Darwish et al., the higher concentrations of cinnamic acid (≥600 mg/L) were found to significantly reduce bio-film formation, extracellular polymeric substance (EPS) production, and overall biomass of single-species and multispecies oral biofilms. Further, chemoinformatic analysis revealed cinnamic acid to be in agreement with the Rule of Five, as established by Lipinski, which contributes to the profile of a drug. These results indicate that cinnamic acid is a promising plant-based compound to treat polymicrobial biofilm-related oral infections, but it should be further validated by peer-reviewed and in vivo research [53].

ADMET simulation also indicates the aptness of these compounds as adjunictive antibiofilm agents. Eugenol and cinnamic acid displayed good gastrointestinal absorption, good oral bioavailability, and better toxicity than chlorhexidine, which is linked to mucosal irritation, dysgeusia, and oral microbiota disturbance with prolonged use [46,48,54]. Their moderate lipophilicity, physicochemical characteristics, and low polar surface area, as well as the lack of predicted hERG inhibition, allow them to be used in topical oral formulations (as mouthrinses and gels).

Combining the docking outcomes with the genetic data provides a consistent model whereby the ENAM rs3796704 A allele could be a predisposing factor to the compromised enamel, which increases the bacterial adherence and maturation of the biofilm. SrtA is a direct contribution in this pathogenic process, as it attaches vital adhesins to the bacterial surface. Here, SrtA eugenol or cinnamic acid inhibition can compensate for the lower enamel resistance with the risk allele, which offers a targeted preventive therapy for genetically predisposed patients.

## 5. Conclusions

This work is a combination of host genetics and in silico virulence targeting, giving a complete picture of caries susceptibility within a cohort of females in Iraq. Analysis of the polymorphism of ENAM rs3796704 has shown that the polymorphism could contribute to the regulation of dental caries vulnerability in individuals. The G allele and GG genotype were inclined to be more common in controls without caries and A allele and AA in dentist patients, indicating the protective effect of G and the risk-promoting effect of A. The allelic distribution and genetic model analysis showed that some of the genotype level associations were not statistically significant, but a biologically plausible role of ENAM rs3796704 in enamel fragility and caries predisposition is suggested. The fact that the Hardy–Weinberg equilibrium in both groups is not in equilibrium is an additional indication that there are non-random genetic effects in this population.

The in silico component is used to support these results by demonstrating that eugenol and cinnamic acid have an ability to bind with the *Streptococcus mutans* Sortase A, with affinities that are a little higher than that of chlorhexidine. Inhibition of the hydro-phobic access channel of the enzyme is mainly caused by eugenol, and the residues in the catalytic region are interacted with by cinnamic acid, which points to two different but complementary inhibitory actions. According to the ADMET predictions, the two phytochemicals have good oral bioavailability and safety profiles that can be used orally with topical application, compared to the adverse effects of prolonged local use of chlorhexidine.

These data combined allow the conceptualization of a model according to which ENAM rs3796704 influences the baseline structural resilience of enamel, and the cariogenic challenge increases the carcinogenicity of vulnerable surfaces under the influence of *S. mutans* SrtA-mediated adhesion. Inhibition of SrtA in phyto-chemicals such as eugenol and cinnamic acid has proven to be a promising approach to reduce bacterial virulence, especially in people who possess risk-based variants of ENAM. The case–control design, single-center recruitment, and the use of docking and predicted pharmacokinetic profiles, however, highlight the need to use larger and multi-ethnic studies and to functionalize the assays, biofilm models and clinical studies of enzymes. The direction to take in future research should be towards genotype- and phytochemical-guided preventive strategies integrating the host genetics, microbial pathogenicity, and targeted control of biofilm formation.

Limitations:

1. A limitation of this study is that the sample size was determined by feasibility recruitment within a single center and a restricted cohort, rather than through an a priori power calculation. Even with our post hoc power consideration, which is dependent on the observed allele frequencies and effect estimates, it would be impossible to prove the association unless larger multi-center cohorts were examined to enhance the confidence interval and allow the performance of subgroup/interaction analyses (e.g., environmental exposures and microbial variables).
2. Though molecular docking and molecular dynamics analyses have been able to give mechanistic data on the ligand-Sortase A interactions, there are no experimental biofilm or enzymatic assays to directly validate the proposed inhibitory effect. In vitro validation in future studies is needed to enhance translational relevance.
3. The case–control design does not allow causal inference, and although the cohort was demographically homogeneous, it may not represent broader population diversity. Docking results require experimental confirmation through SrtA inhibition assays, biofilm studies, and in vivo validation. Despite these limitations, the findings highlight a promising interaction between ENAM genetic variation, *S. mutans* virulence, and phytochemical inhibitors, warranting further investigation into genotype-informed caries prevention strategies.
4. Female respondents were used to minimize biological heterogeneity and the confounding effects of sex in a genetic study. This sex-restricted design, however, discourages a wide-range generalization of the findings to the rest of the population, especially males.
5. The major weakness is that the sample size was feasibility-based and there was no a priori power calculation done. The post hoc power analyses cannot replace prospective sample size planning, and hence the study might not be sufficiently powered to detect small SNP effects, such that the results need to be validated with larger and independent populations.
6. The kappa statistics were not used to determine the formal intra-examiner reliability, though all the examinations were conducted by one experienced examiner under standardized clinical conditions. Furthermore, radiographic examination was not done, and the diagnosis of caries was based on the clinical determined assessment only, which could have resulted in underestimation of interproximal caries and, therefore, low DMFT scores.

## Figures and Tables

**Table 1:** Primers that were designed and used for the study.

ID	Sequence	Molecular Weight (g/mol)	Extinction Coefficient (I/(mol·cm))	Tm (°C)	Annealing Temperature
Primer 1(Forward)	ATTACGGCGACCTGGTCTTC	6084.0	183,100.0	64.9	64.9 °C
Primer 2 (Reverse)	CTCTCTGCCCTTGGGCTTG	5737.8	155,300.0	65.7	

**Table 2:** Thermal profile cycles of HRM genotyping.

Step ID	Temperature (°C)	Time (s)	Cycles
Enzyme activation step	94 (°C)	30	1
Denaturation step	94 (°C)	5	40
Annealing step	60 (°C)	15	
Extension step	72 (°C)	20	
HRM	55–95 (°C)	0.2 s for 1 degree	

**Table 3:** Genotype frequencies of rs3796704 SNP of ENAM gene among tooth decay disease patients and controls.

Genotype	Patient No. (%)	Control No. (%)	Odds Ratio	95% CI	*p* Value
GG	36 (30%)	52 (43.33%)	0.5604	0.2642 to 1.1889	0.1313
GA	72 (60%)	64 (53.33%)	1.3125	0.6364 to 2.7070	0.4616
AA	12 (10%)	4 (3.34%)	3.2222	0.6234 to 16.6559	0.1627
Total	120 (100%)	120 (100%)			

**Table 4:** Allele frequencies of the rs3796704 SNP of the ENAM gene among tooth decay disease patients and controls.

Allele	Patient No. (%)	Control No. (%)	Odds Ratio	95% CI	*p* Value
G	144 (60%)	168 (70%)	0.6429	0.4405 to 0.9382	0.02
A	96 (40%)	72 (30%)	1.5556	1.0659 to 2.2702	

**Table 5:** Genotype frequencies of the rs3796704 SNP of the ENAM gene among tooth decay disease patients and controls in comparison with the Hardy–Weinberg equilibrium (HWE).

Group	GG	GA	AA	X^2^	*p*
Patients	36	72	12	7.37	0.0250
Expected	43.2	57.6	19.2		
Controls	52	64	4		
Expected	58.8	50.4	10.8		

**Table 6:** Genetic model for the rs3796704 SNP association with tooth decay disease.

**Recessive Model**					
**Genotype**	**Patient No.**	**Control No.**	**Odds Ratio**	**95% CI**	***p*** **value**
GG + GA	108	116	0.3103	0.0971 to 0.9915	0.0483
AA	12	4			
**Dominant Model**					
**Genotype**	**Patient No.**	**Control No.**	**Odds Ratio**	**95% CI**	***p*** **value**
GG	36	52	0.5604	0.3293 to 0.9539	0.0328

**Table 7:** Merged interaction table for eugenol and cinnamic acid with Sortase A (4TQX).

	Ligand	Residue	Interaction Type
Eugenol	Ala92	Conventional H-bond	Phenolic -OH
Eugenol	Ala92	van der Waals	Aromatic ring
Eugenol	Ala97	Alkyl	Allyl chain
Eugenol	Val166	π-Alkyl	Aromatic ring
Eugenol	Val168	π-Alkyl	Aromatic ring
Eugenol	Pro151	Alkyl	Allyl chain
Eugenol	Leu110	van der Waals	Aromatic ring
Eugenol	Phe110	van der Waals	Aromatic ring
Eugenol	Tyr318	van der Waals	Aromatic ring
Eugenol	Ala91	Alkyl	Allyl chain
Cinnamic Acid	His120	Conventional H-bond	Carboxyl -COOH
Cinnamic Acid	Arg197	H-bond/Polar	Carboxyl -COOH
Cinnamic Acid	Gly192	van der Waals	Ligand backbone
Cinnamic Acid	Gly193	van der Waals	Ligand backbone
Cinnamic Acid	Val166	Hydrophobic	Phenyl ring
Cinnamic Acid	Leu169	Hydrophobic	Phenyl ring
Cinnamic Acid	Ile182	Hydrophobic	Phenyl ring
Cinnamic Acid	Trp194	π–π stacking	Phenyl ring
Cinnamic Acid	Cys184	Catalytic proximity	Carboxyl -COOH
Cinnamic Acid	His120	Catalytic proximity	Carboxyl -COOH

**Table 8:** Highest binding affinity values of eugenol, cinnamic acid, and chlorhexidine against *S. mutans* Sortase A (4TQX).

Ligand	Smiles	Best Binding Affinity (kcal/mol)
Eugenol	COC1=C(O)C=CC(CC=C)=C1	−4.961
Cinnamic Acid	OC(=O)C=CC1=CC=CC=C1	−4.939
Chlorhexidine (Positive Control)	C1=CC(=CC=C1N/C(=N/C(=NCCCCCCN=C(/N=C(/NC2=CC=C(C=C2)Cl)\N)N)N)/N)Cl	−4.692

**Table 9:** Unified ADMET comparison table (eugenol vs. cinnamic acid vs. chlorhexidine), produced using ADMET–AI.

Category	Property	Eugenol	Cinnamic Acid	Chlorhexidine	Units
Physicochemical	Molecular Weight	164.20	148.16	505.46	Dalton
	LogP	2.13	1.78	3.34	log-ratio
	H-Bond Acceptors	2	1	2	#
	H-Bond Donors	1	1	6	#
	Lipinski RO5	4	4	2	# of 4
	QED	0.69	0.65	0.17	–
	Stereo Centers	0	0	0	#
	TPSA	29.46	37.30	177.58	Å^2^
Absorption	Human Intestinal Absorption	1.00	1.00	0.85	–
	Oral Bioavailability	0.44	0.92	0.66	–
	Aqueous Solubility	–2.06	–2.24	–3.40	log(mol/L)
	Lipophilicity	2.54	–0.39	1.04	log-ratio
	Hydration Free Energy	–6.39	–8.67	–8.01	kcal/mol
	Cell Permeability	–4.55	–4.54	–5.84	log(10^−6^ cm/s)
	PAMPA Permeability	0.86	0.35	0.47	–
	P-gp Inhibition	0.04	3.69 × 10^−3^	0.67	–
Distribution	BBB Penetration	0.77	0.80	0.59	–
	Plasma Protein Binding	80.45%	92.79%	84.67%	%
	Volume of Distribution	0.00	4.34	22.55	L/kg
Metabolism	CYP1A2 Inhibition	0.67	0.02	0.22	–
	CYP2C19 Inhibition	0.46	0.02	0.31	–
	CYP2C9 Inhibition	0.11	0.02	0.08	–
	CYP2D6 Inhibition	0.12	0.01	0.83	–
	CYP3A4 Inhibition	0.27	1.54 × 10^−4^	0.16	–
	CYP2C9 Substrate	0.15	0.45	0.08	–
	CYP2D6 Substrate	0.29	0.02	0.38	–
	CYP3A4 Substrate	0.38	0.09	0.71	–
Excretion	Half-Life	0.00	0.00	55.73	h
	Hepatocyte Clearance	115.18	0.00	39.50	μL/min/10^6^ cells
	Microsomal Clearance	64.79	4.54	16.20	μL/min/mg
Toxicity	hERG Blocking	0.07	3.03 × 10^−3^	0.90	–
	Clinical Toxicity	2.56 × 10^−3^	0.14	0.20	–
	Mutagenicity	0.14	0.01	0.26	–
	Drug-Induced Liver Injury	0.09	0.85	0.43	–
	Carcinogenicity	0.11	0.26	0.34	–
	Acute Toxicity (LD50)	1.94	1.60	2.53	log(1/(mol/kg))
	Skin Reaction	0.85	0.51	0.56	–
	Androgen Receptor (FL)	0.01	0.07	0.02	–
	Androgen Receptor (LBD)	1.30 × 10^−3^	0.01	0.01	–
	Aryl Hydrocarbon Receptor	0.05	4.13 × 10^−3^	0.20	–
	Aromatase	0.01	2.66 × 10^−4^	0.04	–
	Estrogen Receptor (FL)	0.07	0.07	0.07	–
	Estrogen Receptor (LBD)	0.02	0.01	0.03	–
	PPAR-γ	0.01	0.02	0.01	–
	Nrf2/ARE	0.18	0.09	0.19	–
	ATAD5	0.01	0.01	0.03	–
	HSF Response	0.08	0.01	0.06	–
	Mitochondrial Membrane Potential	0.07	1.56 × 10^−3^	0.18	–
	Tumor Protein p53	0.02	2.28 × 10^−3^	0.15	–

# represents the number of respective molecular features.

**Table 10:** Comparison of SwissTargetPrediction profiles of eugenol, cinnamic acid, and chlorhexidine.

No.	Target (Common Name)	Gene	Target Class	Eugenol Probability	Cinnamic Acid Probability	Chlorhexidine Probability
1	Fatty acid desaturase 1	*FADS1*	Enzyme	0.1334	–	–
2	Histone deacetylase 6	*HDAC6*	Epigenetic eraser	0.1334	0.0000	0.0000
3	EglN1	*EGLN1*	Oxidoreductase	0.1251	–	–
4	VEGF-A	*VEGFA*	Secreted protein	0.1251	–	–
5	Carbonic anhydrase II	*CA2*	Lyase	0.1251	0.1399	–
6	Carbonic anhydrase I	*CA1*	Lyase	–	0.1399	–
7	GPR84	*GPR84*	GPCR (Family A)	0.1251	–	–
8	COX-1	*PTGS1*	Oxidoreductase	0.1251	–	–
9	PARP-1	*PARP1*	Enzyme	0.1251	–	–
10	SRC kinase	*SRC*	Kinase	0.1251	–	–
11	Adenosine A1 receptor	*ADORA1*	GPCR A	0.1251	–	–
12	Adenosine A2A receptor	*ADORA2A*	GPCR A	0.1251	–	–
13	Steroid 5-α reductase 1	*SRD5A1*	Oxidoreductase	0.1251	–	–
14	IL-8 receptor B (CXCR2)	*CXCR2*	GPCR A	0.1251	–	–
15	Hydroxycarboxylic acid receptor 2	*HCAR2*	GPCR A	–	0.8870	–
16	Aldose reductase	*AKR1B1*	Enzyme	–	0.1303	–
17	Monocarboxylate transporter 1	*SLC16A1*	Transporter	–	0.1012	–
18	Toll-like receptor 4	*TLR4*	TLR receptor	–	0.0918	–
19	Carbonic anhydrase VII	*CA7*	Lyase	–	0.0823	–
20	Carbonic anhydrase XIV	*CA14*	Lyase	–	0.0823	–
21	Carbonic anhydrase IX	*CA9*	Lyase	–	0.0823	–
22	Carbonic anhydrase VB	*CA5B*	Lyase	–	0.0823	–
23	Carbonic anhydrase VA	*CA5A*	Lyase	–	0.0823	–
24	TRPA1 ion channel	*TRPA1*	Voltage-gated ion channel	–	0.0727	–
25	ALOX5	*ALOX5*	Oxidoreductase	–	0.0535	–
26	MMP9	*MMP9*	Protease	–	0.0535	–
27	MMP1	*MMP1*	Protease	–	0.0535	–
28	MMP2	*MMP2*	Protease	–	0.0535	–
29	PTP1B	*PTPN1*	Phosphatase	–	0.0535	–
30	5-HT2B receptor	*HTR2B*	GPCR A	–	–	0.1202
31	Thrombin	*F2*	Protease	–	–	0.0000
32	BCHE	*BCHE*	Hydrolase	–	–	0.0000
33	ACHE	*ACHE*	Hydrolase	–	–	0.0000
34	Beta-secretase 1 (BACE1)	*BACE1*	Protease	–	–	0.0000
35	DHFR	*DHFR*	Oxidoreductase	–	–	0.0000
36	Dopamine D2 receptor	*DRD2*	GPCR A	–	–	0.0000
37	CCR5	*CCR5*	GPCR A	–	–	0.0000
38	AKT1	*AKT1*	Kinase	–	–	0.0000
39	IDO1	*IDO1*	Enzyme	–	0.0439	0.0000
40	BACE1	*BACE1*	Protease	–	–	0.0000

## Data Availability

All the data supporting our findings are contained within the manuscript.

## Supplementary Materials

The following supporting information can be downloaded at https://www.mdpi.com/article/10.3390/dj14060360/s1. Figure S1: Original image of analysis of genomic DNA samples extracted by agarose gel electrophoresis (1% w/v).
