# Supplementary figures and images for: Integrative Analysis of ENAM rs3796704 Polymorphism and Eugenol–Cinnamic Acid Docking/ADMET Against Biofilm-Forming Streptococcus Mutans: Genetic–Phytochemical Links to Oral Dysbiosis
Source: Dent J (Basel). 2026 Jun 11;14(6):360. doi: 10.3390/dj14060360 (PMC13298706; doi:10.3390/dj14060360)

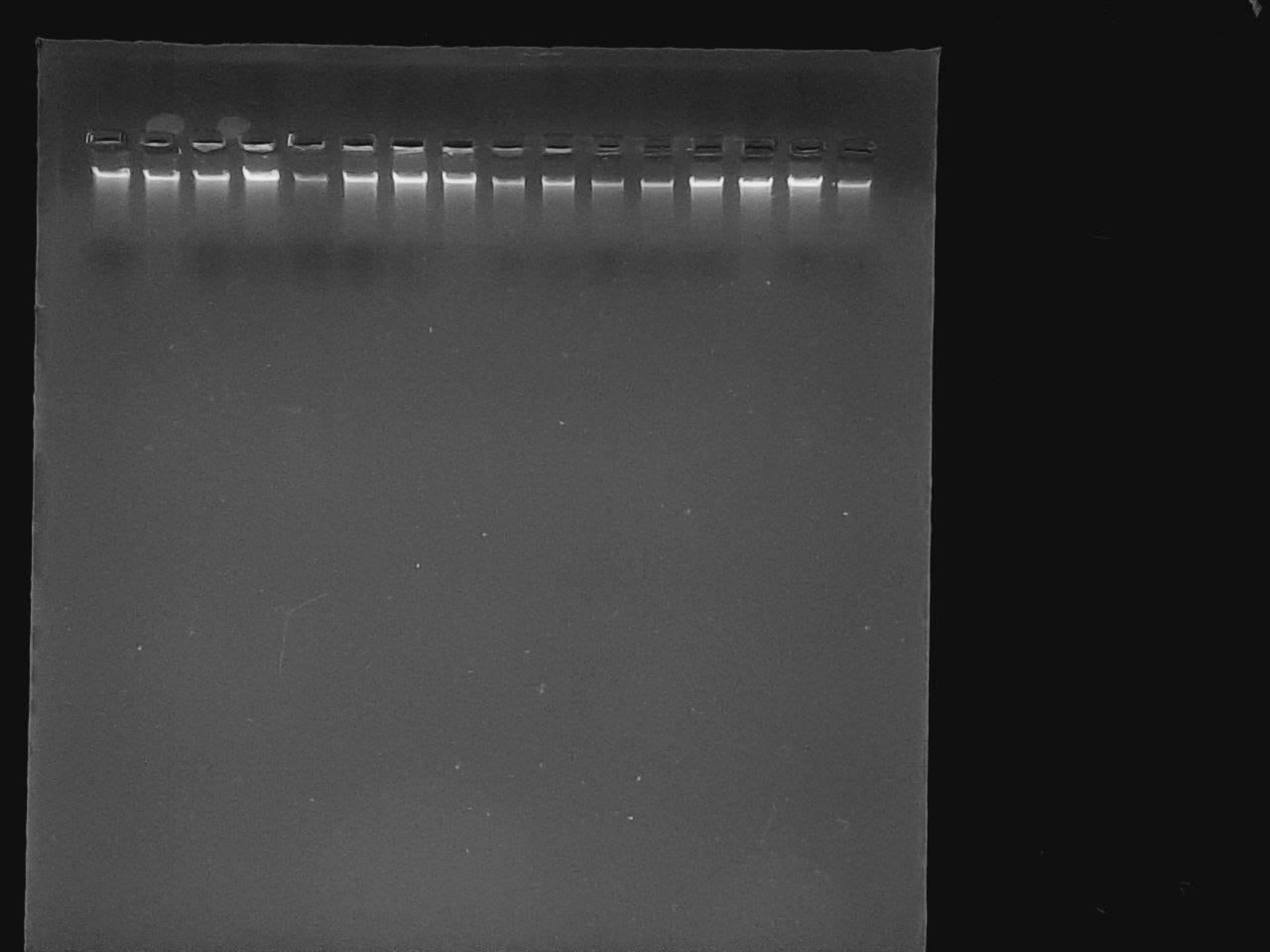

Supplement: Supplementary file 1 [file dentistry-14-00360-s001.zip › dentistry-4045620-supplementary.jpg]
